# Supplementary figures and images for: Comparing the efficiency of open and enclosed filtration systems in environmental DNA quantification for fish and jellyfish
Source: PLoS One. 2020 Apr 20;15(4):e0231718. doi: 10.1371/journal.pone.0231718 (PMC7170242; doi:10.1371/journal.pone.0231718)

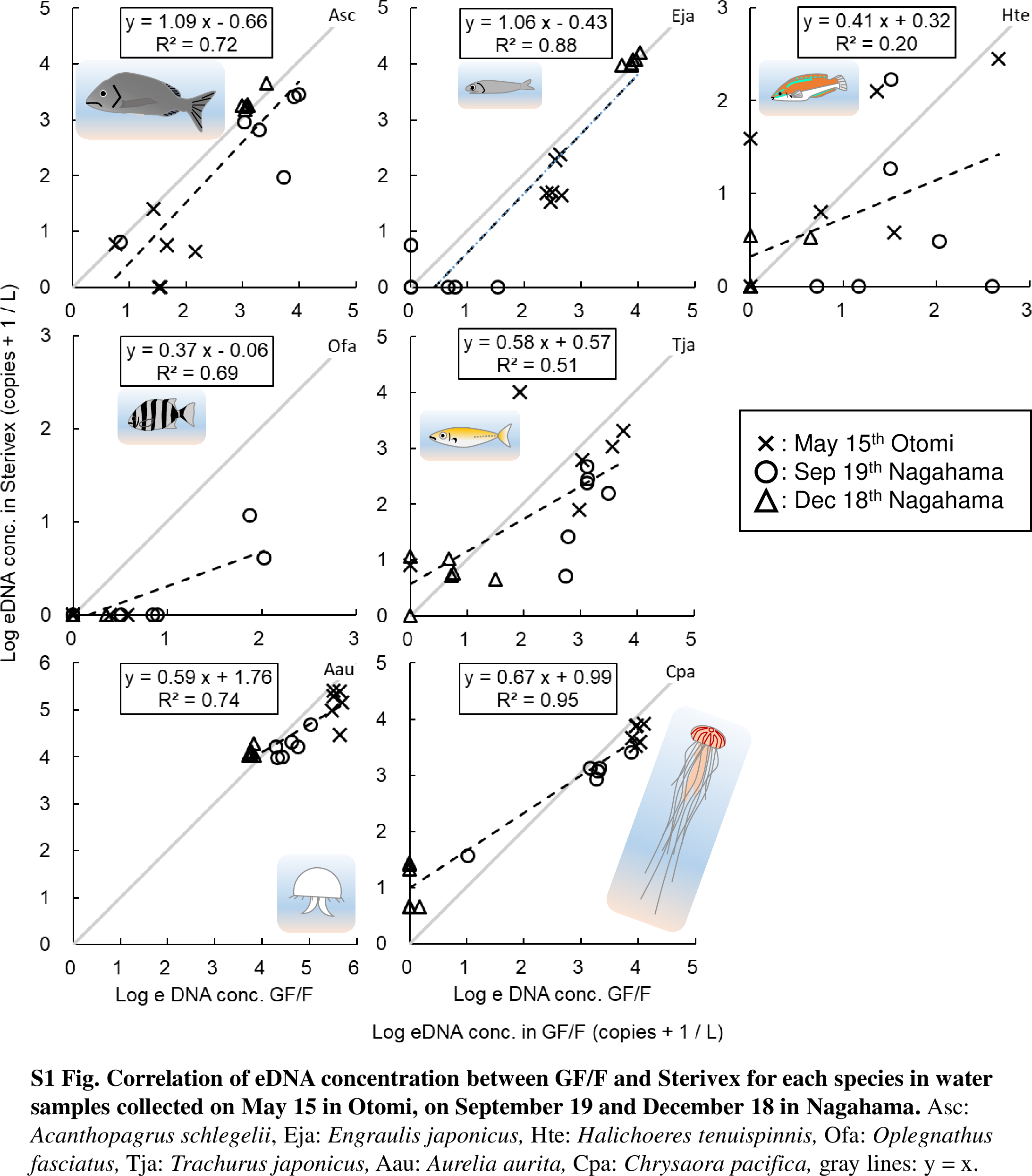

Supplement: S1 Fig — Asc: Acanthopagrus schlegelii, Eja: Engraulis japonicus, Hte: Halichoeres tenuispinnis, Ofa: Oplegnathus fasciatus, Tja: Trachurus japonicus, Aau: Aurelia aurita, Cpa: Chrysaora pacifica, gray lines: y = x. (TIF) [file pone.0231718.s005.tif]

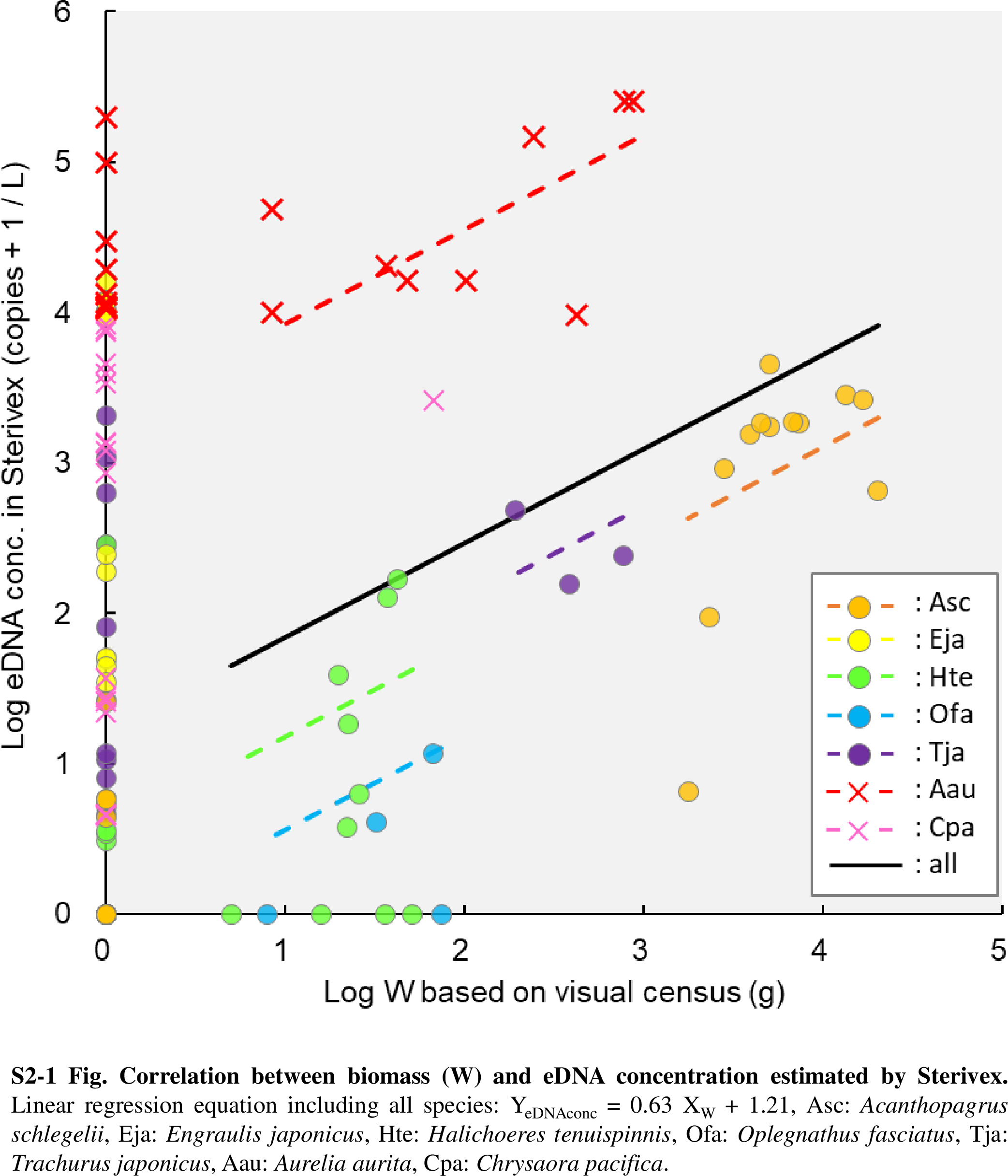

Supplement: S2 Fig — Linear regression equation including all species: YeDNAconc = 0.63 XW + 1.21, Asc: Acanthopagrus schlegelii, Eja: Engraulis japonicus, Hte: Halichoeres tenuispinnis, Ofa: Oplegnathus fasciatus, Tja: Trachurus japonicus, Aau: Aurelia aurita, Cpa: Chrysaora pacifica. (TIF) [file pone.0231718.s006.tif]

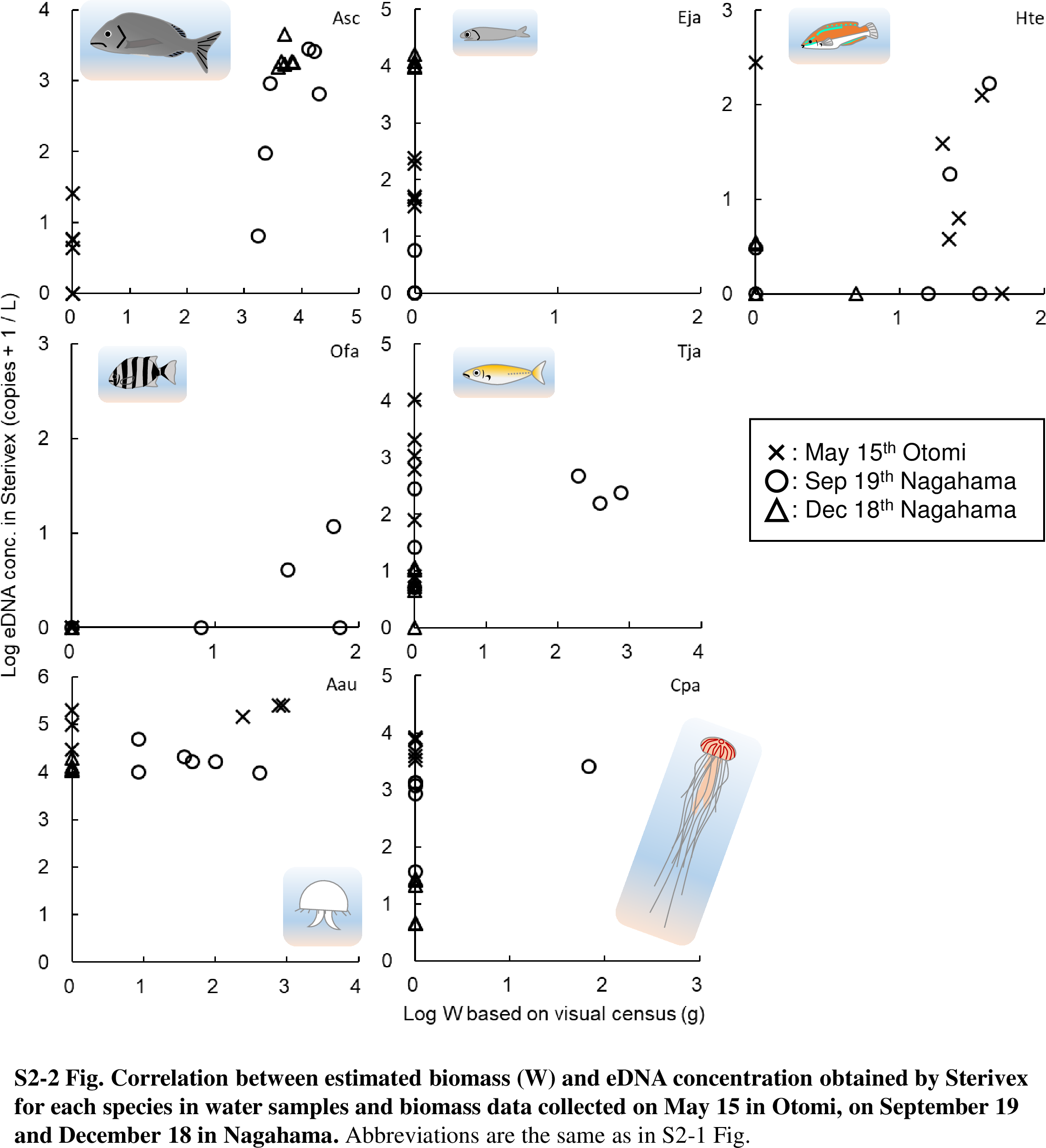

Supplement: S3 Fig — Abbreviations are the same as in S2 Fig. (TIF) [file pone.0231718.s007.tif]

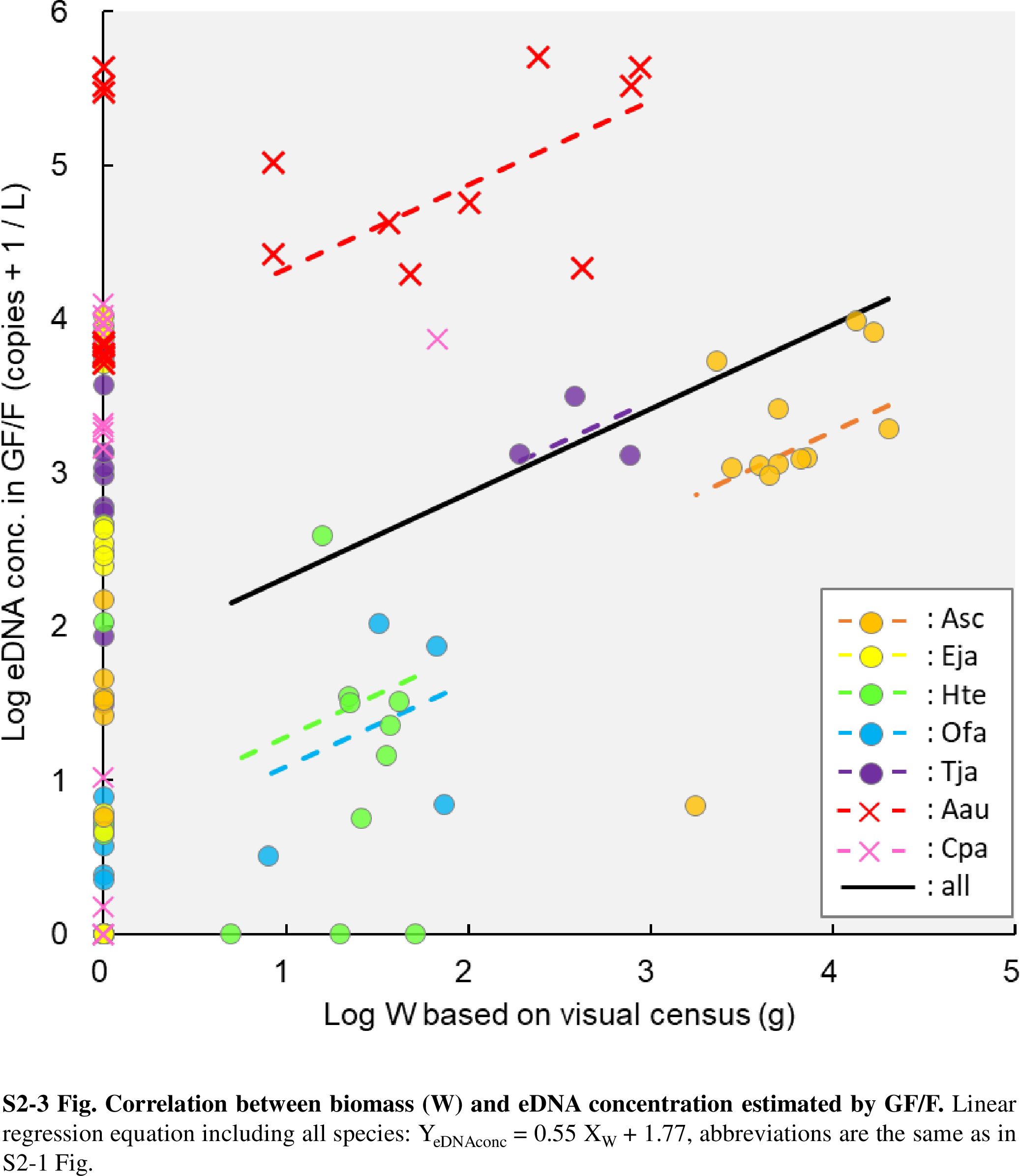

Supplement: S4 Fig — Linear regression equation including all species: YeDNAconc = 0.55 XW + 1.77, abbreviations are the same as in S2 Fig. (TIF) [file pone.0231718.s008.tif]

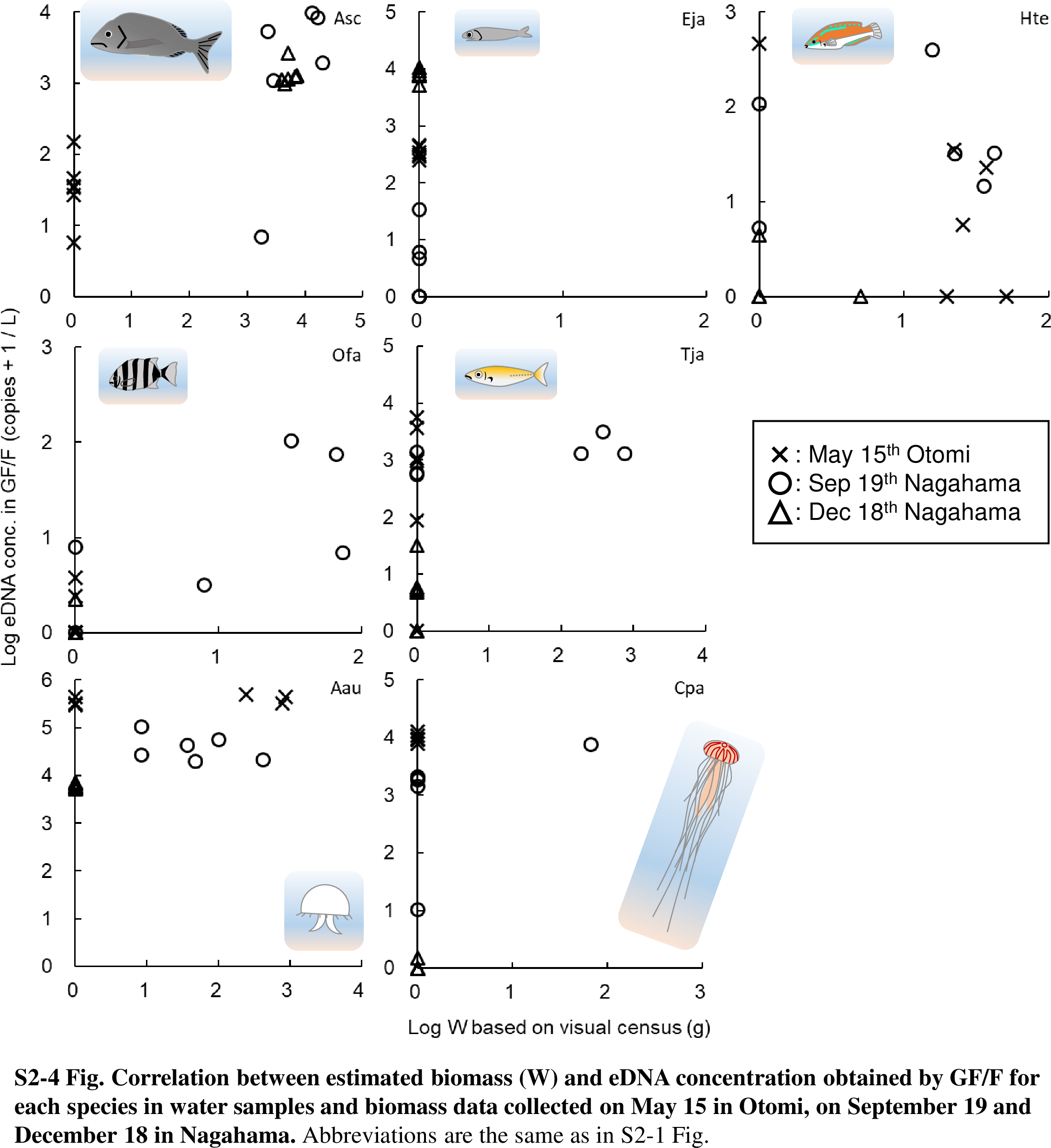

Supplement: S5 Fig — Abbreviations are the same as in S2 Fig. (TIF) [file pone.0231718.s009.tif]
