# Supplementary material for: Comparing the efficiency of open and enclosed filtration systems in environmental DNA quantification for fish and jellyfish
Source: PLoS One. 2020 Apr 20;15(4):e0231718. doi: 10.1371/journal.pone.0231718 (PMC7170242; doi:10.1371/journal.pone.0231718)
Supplement: Attachment — Submitted filename: Response to Reviewers_2.docx [file pone.0231718.s011.docx]

***Response and our reply were shown in boldfaced type.**

PONE-D-19-32313R1

Comparing the efficiency of open and enclosed filtration systems in environmental DNA quantification for fish and jellyfish

PLOS ONE

Dear Dr. Takahashi,

Thank you for submitting your manuscript to PLOS ONE. After careful consideration, we feel that it has merit but does not fully meet PLOS ONE’s publication criteria as it currently stands. Therefore, we invite you to submit a revised version of the manuscript that addresses the points raised during the review process.

Authors need to make minor corrections in accordance with the comments of reviewer #3 before the manuscript is accepted.

We would appreciate receiving your revised manuscript by May 03 2020 11:59PM. When you are ready to submit your revision, log on to https://www.editorialmanager.com/pone/ and select the 'Submissions Needing Revision' folder to locate your manuscript file.

If you would like to make changes to your financial disclosure, please include your updated statement in your cover letter.

To enhance the reproducibility of your results, we recommend that if applicable you deposit your laboratory protocols in protocols.io, where a protocol can be assigned its own identifier (DOI) such that it can be cited independently in the future. For instructions see: http://journals.plos.org/plosone/s/submission-guidelines#loc-laboratory-protocols

Please include the following items when submitting your revised manuscript:

A rebuttal letter that responds to each point raised by the academic editor and reviewer(s). This letter should be uploaded as separate file and labeled 'Response to Reviewers'.

A marked-up copy of your manuscript that highlights changes made to the original version. This file should be uploaded as separate file and labeled 'Revised Manuscript with Track Changes'.

An unmarked version of your revised paper without tracked changes. This file should be uploaded as separate file and labeled 'Manuscript'.

Please note while forming your response, if your article is accepted, you may have the opportunity to make the peer review history publicly available. The record will include editor decision letters (with reviews) and your responses to reviewer comments. If eligible, we will contact you to opt in or out.

We look forward to receiving your revised manuscript.

Kind regards,

Ruslan Kalendar, PhD

Academic Editor

PLOS ONE

Reviewers' comments:

Reviewer's Responses to Questions

Comments to the Author

1. If the authors have adequately addressed your comments raised in a previous round of review and you feel that this manuscript is now acceptable for publication, you may indicate that here to bypass the “Comments to the Author” section, enter your conflict of interest statement in the “Confidential to Editor” section, and submit your "Accept" recommendation.

Reviewer #2: All comments have been addressed

Reviewer #3: All comments have been addressed

2. Is the manuscript technically sound, and do the data support the conclusions?

Reviewer #2: Yes

Reviewer #3: Yes

3. Has the statistical analysis been performed appropriately and rigorously?

Reviewer #2: Yes

Reviewer #3: Yes

4. Have the authors made all data underlying the findings in their manuscript fully available?

Reviewer #2: Yes

Reviewer #3: Yes

5. Is the manuscript presented in an intelligible fashion and written in standard English?

Reviewer #2: Yes

Reviewer #3: Yes

6. Review Comments to the Author

Reviewer #3:

Thank you for allowing me to re-review your manuscript, I feel that it is much improved. I have compiled a list of suggestions and comments below, however I will leave these suggestions up to the authors and/or editor to implement at your discretion as most of the comments are wording/style suggestions and small mistakes, all of which should be very easily remedied. The line numbers presented below refer to the line numbers on the revised manuscript.

**(Response)**

**Thank you for your constructive comments. We revised our manuscript as per your suggestions. We consider that it was substantially improved.**

Line 34 “was relatively low. Concentration of eDNA correlated” insert ‘The’ at the beginning of the sentence “was relatively low. The concentration of eDNA correlated”

**Our reply: As per your suggestion, we inserted ‘The’ at the beginning of the sentence (Line 34).**

Line 54-55 “and tanks [11, 12]. Concentration of eDNA is also positively correlated with” reword by inserting The and change is to was. “and tanks [11, 12]. The concentration of eDNA was also positively correlated with”

**Our reply: As per your suggestion, we revised “and tanks [11, 12]. Concentration of eDNA is also positively correlated with” as “and tanks [11, 12]. The concentration of eDNA was also positively correlated with” (Line 54-55).**

Line 81-82 “when using an enclosed, as opposed to an open, filter to detect fish species in ponds [39].” Reword and repunctuate to “when using an enclosed filter, as opposed to an open filter, to detect fish species in ponds [39].”

**Our reply: As per your suggestion, we revised “when using an enclosed, as opposed to an open, filter to detect fish species in ponds [39].” as “when using an enclosed filter, as opposed to an open filter, to detect fish species in ponds [39].” (Line 81-82).**

Line 85-86 “Especially in the eDNA metabarcoding method that utilizes MiFish PCR primers, the number of species” Reword “This may be particularly true when using the eDNA metabarcoding method that utilizes MiFish PCR primers, whereby the number of species”

**Our reply: As per your suggestion, we revised “Especially in the eDNA metabarcoding method that utilizes MiFish PCR primers, the number of species” as “This may be particularly true when using the eDNA metabarcoding method that utilizes MiFish PCR primers, whereby the number of species” (Line 82-84).**

Line 97-98 “filtration methods will clarify such differences between them. Based on such knowledge, adaptive usage” suggested reword so that “such” is not used twice in a row.

**Our reply: As per your suggestion, we removed the first “such” and revised “such differences” as “the differences” (Line 97).**

Line 131 If you are going to go with the argument of reporting salinity as unit-less, and you are using a conductivity probe, you still need to state what you are reporting in the methods. I suggest “salinity (measured by a water quality meter with a conductivity probe and reported in practical salinity units (psu): LAQUAact ES-71, Horiba” and then the values of salinity reported throughout the document do not require units.

**Our reply: As per your suggestion, we revised “salinity (measured by a water conductivity meter with a probe: LAQUAact ES-71, Horiba” as “salinity (measured by a water quality meter with a conductivity probe and reported in practical salinity units (psu): LAQUAact ES-71, Horiba” (Line 131-132).**

Line 143-145 I suggest adding a little more detail to explain the inclusion of the picture and I agree with the response you provided in your rebuttal that it should be included. I suggest rewording this “filtration using GF/F and Sterivex. Illustration depicted two species of jellyfish (Aau and Cpa), pelagic fish (Eja and Tja) and demersal fish (Ofa, Hte and Asc). Seawater samples,” and adding this information “filtration using GF/F and Sterivex. The illustration depicts two species of jellyfish (Aau and Cpa), pelagic fish (Eja and Tja) and demersal fish (Ofa, Hte and Asc) and highlights a visual representation of the position in the water column and schooling behaviour of some of these species assemblages during a typical transect. Seawater samples,” or any similar information as this will point out the importance of this illustration.

**Our reply: Thank you for agreeing with us on that. As per your suggestion, we added a little more detail. We revised “filtration using GF/F and Sterivex. Illustration depicted two species of jellyfish (Aau and Cpa), pelagic fish (Eja and Tja) and demersal fish (Ofa, Hte and Asc). Seawater samples,” as “filtration using GF/F and Sterivex. The illustration depicts two species of jellyfish (Aau and Cpa), pelagic fish (Eja and Tja) and demersal fish (Ofa, Hte and Asc) and highlights a visual representation of the position in the water column and schooling behaviour of some of these species assemblages during a typical transect. Seawater samples,” (Line 144-148).**

Line 184 Check this salinity value (2.80) as this would be freshwater “respectively. Salinity near pier 2 was 2.80, and visibility was about 1 m near the surface” I think it is 28.0.

**Our reply: Thank you for finding our mistake. We revised “2.80” as “28.0” (Line 187).**

Line 370 change “ware” to “were”

**Our reply: It is also our mistake. Thank you for finding it. We revised “ware” as “were” (Line 373).**

Paragraph Line 452-477 is not presented correctly, has many minor wording mistakes, and the section that was added Line 458-474 does not fit well. I will try and rework a suggestion below, however this is only a suggestion.

Regarding explanation iii), extraction losses may offer a possible explanation as to why the eDNA concentrations obtained by the two filtration methods were different. It is known that the size distribution of eDNA particles varies with environmental conditions [40]. The amount of detected eDNA depends on different eDNA characteristics (size, spatial structure, extra- and intracellular, and particle-bound and free) [30], indicating that the optimal filter choice varies for different extraction methods and there are different protocol combinations suitable for different organisms [28, 58]. At the time of extraction, it is necessary for DNA to flow out of cells, and be removed from filter paper by elution and centrifugation, so that it does not remain on the walls of the tube and column, resulting in eDNA loss.

Furthermore, the procedure of eDNA extraction using the GF/F and Sterivex filtration method was similar, but not exactly the same, during the experiments presented here, which was a necessity to compare the two methods (Fig 2). Therefore it possible that these differences in methods may contribute to differences detected in the results. Specifically, BAC was used for DNA preservation of the GF/F samples, and RNAlater was used for DNA preservation of the Sterivex filtrations. BAC is cationic surfactant and reduces microbial activity [59], whereas RNAlater is a stable reagent which deactivates 463 nucleases [60]. Water samples with BAC are reported to retain more than 92% of fish DNA for 8-h at ambient temperature [59], and those with RNAlater are reported to successfully preserve the same amount of planktonic DNA for over 1 month at ambient temperature as frozen samples [60]. This study conducted the process from sampling to preservation in a freezer in less than seven hours, and the samples were transported on ice immediately after the addition of BAC or RNAlater. Therefore, we assume that there was not much difference in the result due to the preservation step. However, it is also reported that fish species detected by metabarcoding analysis using BAC were lower than those stored on ice [61], whereas RNAlater yielded a substantial precipitate that inhibited qPCR amplification of fish [62]. RNAlater has been shown to store good quality DNA, but not always in high enough quantities for metabarcoding analysis of marine organisms [63]. Therefore, the difference between BAC and RNAlater preservation may be a subject that requires further investigation.

**Our reply: We are in agreement with your view. We moved the last sentence to the original place, and revised as per your suggestion (Line 455-483).**

Line 488 “seawater fish” should be changed to “marine fish”

**Our reply: As per your suggestion, we revised “seawater fish” as “marine fish” (Line 494).**

I sincerely hope these suggestions are helpful, and please implement them at your discretion, especially the suggested paragraph edit above.

**(Response)**

**Your suggestions were really helpful for us. Thank you so much.**

7. PLOS authors have the option to publish the peer review history of their article (what does this mean?). If published, this will include your full peer review and any attached files.

If you choose “no”, your identity will remain anonymous but your review may still be made public.

Do you want your identity to be public for this peer review? For information about this choice, including consent withdrawal, please see our Privacy Policy.

Reviewer #2: No

Reviewer #3: No

[NOTE: If reviewer comments were submitted as an attachment file, they will be attached to this email and accessible via the submission site. Please log into your account, locate the manuscript record, and check for the action link "View Attachments". If this link does not appear, there are no attachment files to be viewed.]

While revising your submission, please upload your figure files to the Preflight Analysis and Conversion Engine (PACE) digital diagnostic tool, https://pacev2.apexcovantage.com/. PACE helps ensure that figures meet PLOS requirements. To use PACE, you must first register as a user. Registration is free. Then, login and navigate to the UPLOAD tab, where you will find detailed instructions on how to use the tool. If you encounter any issues or have any questions when using PACE, please email us at figures@plos.org. Please note that Supporting Information files do not need this step.

In compliance with data protection regulations, you may request that we remove your personal registration details at any time. (Remove my information/details). Please contact the publication office if you have any questions.
